# Saleability of anti-malarials in private drug shops in Muheza, Tanzania: a baseline study in an era of assumed artemisinin combination therapy (ACT)

**DOI:** 10.1186/1475-2875-10-238

**Published:** 2011-08-15

**Authors:** Frank M Ringsted, Isolide S Massawe, Martha M Lemnge, Ib C Bygbjerg

**Affiliations:** 1 Department of International Health, Immunology and Microbiology (ISIM), Faculty of Health Sciences, University of Copenhagen, Øster Farimagsgade 5, DK 1014 Copenhagen K, Denmark; 2 National Institute for Medical Research (NIMR), Tanga Centre, P.O. Box 5004, Tanga Tanzania

## Abstract

**Background:**

Artemether-lumefantrine (ALu) replaced sulphadoxine-pymimethamine (SP) as the official first-line anti-malarial in Tanzania in November 2006. So far, artemisinin combination therapy (ACT) is contra-indicated during pregnancy by the national malaria treatment guidelines, and pregnant women depend on SP for Intermittent Preventive Treatment (IPTp) during pregnancy. SP is still being dispensed by private drug stores, but it is unknown to which extent. If significant, it may undermine its official use for IPTp through induction of resistance.

The main study objective was to perform a baseline study of the private market for anti-malarials in Muheza town, an area with widespread anti-malarial drug resistance, prior to the implementation of a provider training and accreditation programme that will allow accredited drug shops to sell subsidized ALu.

**Methods:**

All drug shops selling prescription-only anti-malarials, in Muheza town, Tanga Region voluntarily participated from July to December 2009. Qualitative in-depth interviews were conducted with owners or shopkeepers on saleability of anti-malarials, and structured questionnaires provided quantitative data on drugs sales volume.

**Results:**

All surveyed drug shops illicitly sold SP and quinine (QN), and legally amodiaquine (AQ). Calculated monthly sale was 4,041 doses, in a town with a population of 15,000 people. Local brands of SP accounted for 74% of sales volume, compared to AQ (13%), QN (11%) and ACT (2%).

**Conclusions:**

In community practice, the saleability of ACT was negligible. SP was best-selling, and use was not reserved for IPTp, as stipulated in the national anti-malarial policy.

It is a major reason for concern that such drug-pressure in the community equals *de facto *intermittent presumptive treatment. In an area where SP drug resistance remains high, unregulated SP dispensing to people other than pregnant women runs the risk of eventually jeopardizing the effectiveness of the IPTp strategy.

Further studies are recommended to find out barriers for ACT utilization and preference for self-medication and to train private drug dispensers.

## Background

Anti-malarials have been widely available in private shops for home-treatment for decades [1]. In Tanzania drug shops known as *duka la dawa baridi *(DLDB), although formally only licensed to sell over-the-counter (OTC) drugs, frequently sell prescription-only anti-malarials for treatment of fever or malaria, and are important providers, often staffed by personnel with little or some medical training, working in rural market centres where licensed pharmacies are lacking[2]. At present only one anti-malarial, amodiaquine (AQ), has the status as an OTC drug, but is no longer recommended; all other anti-malarials are prescription-only drugs [3].

A national programme aims to upgrade and accredit existing DLDBs into "accredited drug dispensing outlet" (ADDOs) [4], to ensure that more than 80% of rural/peri-urban areas in Tanzania mainland get opportunity to purchase quality basic medicines from properly operated private medicine outlets, manned by trained personnel after provider training. The ADDO programme has the potential for being an important tool in the regulation of private drugs sale, legalizing the sale of first-line drugs, and for enforcing the national anti-malarial drug policy in the private sector at the local level.

Tanzania revised its drug policy in 2006, for artemether-lumefantrine (ALu) to replace SP as first-line treatment for uncomplicated malaria [5], in line with guidelines of the World Health Organization (WHO), which recommends artemisinin combination therapy (ACT) in areas with resistance to previous first-line anti-malarials [6]. With this policy, use of SP is recommended only as intermittent preventive treatment during pregnancy (IPTp). Quinine (QN) has replaced AQ as second-line treatment, and is further indicated as first-line treatment for children weighing less than five kilo and their lactating mothers, and during first trimester of pregnancy. Use of AQ is only indicated where no better drug is available [5], except in Zanzibar, where artesunate-AQ is first-line treatment.

In Tanzania mainland, ALu is provided to all governmental health care facilities (subsidized by Global Fund support), and is marketed in the media as *dawa mseto*; "combination drug".

The effects of this change in policy on the private market for anti-malarials have been studied in districts already covered by the ADDO programme, which so far has been implemented only in the Morogoro, Mtwara, Rukwa and Ruvuma Regions [7]. But little is known of the situation in ordinary DLDBs in the market centres elsewhere in Tanzania, and if the costly ACT may be saleable.

A prior transition in anti-malarial drug policy in 2001, changing the first-line treatment from chloroquine (CQ) to SP, was initially met with negative perceptions by the public, who feared adverse reactions reported in the mass media, and considered the new drug and its single-dose regimen to be too strong for them. Others found SP to be ineffective compared to CQ, which cleared fever more rapidly [8-12]. Thus, guidelines may not be known nor adapted to quickly, particularly not in private drug distribution [13,14]. Recent indications from the study area suggest that SP may still be widely used, as also indirectly reflected in most molecular markers of resistance remaining high (D. Ishengoma, personal communication).

The study objective was, therefore, to perform a baseline study of the private market for anti-malarials in Muheza town, after the malaria policy change from SP to ALu, prior to the implementation of a provider training and accreditation programme that will allow accredited drug shops to sell subsidized artemether-lumefantrine for home-treatment. The paper describes the sale of anti-malarials in private drug shops in Muheza, prior to the anticipated implementation in Tanga Region of the ADDO programme (scheduled to 2009 but delayed).

## Methods

### Study area

The district capital Muheza, the research site, is located in Tanga Region at the main road connecting Mombasa, Tanga and Dar es Salaam, with 15,000 inhabitants (in 2002) living in the three wards that comprise the district town centre [15]. Muheza district was until recently a high-transmission area for malaria, and with high levels of resistance to anti-malarials, including SP [16].

Anti-malarials are within reach for most people living in town or visiting on twice weekly market days. Muheza is one of the 13 districts in Tanzania where the district hospital is a designated mission hospital (Teule); generally well supplied and well staffed, based on cost sharing. The government health facility consists of an old dispensary with no laboratory facilities. This serves as the only public primary health care facility in town, it offers free services but is mostly bypassed by patients, who instead tend to go to the district hospital out-patient department (OPD) where cost sharing is moderate, or to one of the two private clinics or use home-treatment with drugs bought from shops [17]. Since 2004, the number of private drug shops in Muheza town has grown from five drug shops in 2004, to 20 in 2009, including the pharmacies of two private clinics.

### Study methods

Qualitative baseline data were collected on the provider perspectives on the saleability of the drugs presently offered for illicit sale, and actual sale was assessed quantitatively, by means of in-depth open-ended interviews and structured questionnaires respectively

### Study-group and interviews

The study, conducted from July to December 2009, covered the drug shops *(duka la dawa baridi; *DLDB) in Muheza town and the pharmacies of the two private clinics that were all already selling prescription-only anti-malarials and antibiotics, i.e. 20 private drug-outlets in total. Weekly observations and informal in-depth interviews in Kiswahili language, based on question guides with shopkeepers, were the main methods throughout the study, from July to October 2009, combined with a follow-up survey in December of actual drugs sale utilizing a structured questionnaire in Kiswahili.

Initially consent to participate and allow observations and interviews in shops was obtained from all twenty drug-shop owners in town, and from shopkeepers agreeing to be interviewed. The term shopkeeper here refers to the person operating the shop, whether employee or self-employed. Shopkeepers of 16 shops participated throughout the study, including the drug sales survey, whereas two shop-owners withdrew consent, one owner closed one of his two shops during the study period and another had temporarily closed down during the final phase of the study.

The *saleability *of drug - their qualities that allowed them to be sold or their perceived "value" to the consumer as this was understood by the shopkeeper - was explored by qualitative research methods i.e. ethnographic interviews. This included sessions of informal in-depth interviews and observations (seven per shop until no new information was derived); question guides were prepared focusing on prices and how shopkeepers explained their customers' preferences or ambivalences towards the drugs they could buy in the shops. Qualitative interview data consisted of verbatim notes taken in Kiswahili while conducting the interviews. Data analysis was exploratory, unassisted by computer software, identifying the key notions and concepts presented in the paper.

Actual *sale*; the drug sales volume, was calculated based on a survey using two structured questionnaire interviews conducted 14 days apart, from 1^st^- 23^rd^. December 2009, after the short rainy season in November during which malaria transmission is somehow average for the year; in-between the dry season's low transmission and the long rainy season's high transmission. Questionnaire data included initial stock levels for each anti-malarial, date of opening the drug container/box, date of emptying it/the stock level at revisit, and, if drugs had been moved for other reasons than sale. Dates were mostly based on shop registries, as reported by shopkeepers. Counting of tablets, bottles or vials was performed by the shopkeeper, observed by the interviewer, after which monthly sale was calculated in units of adult doses (except syrups, which were calculated as child doses). Drug sale survey questionnaire data were processed in Excel^®^. To estimate the number of infants accessing town, hospital immunization numbers was used as a proxy, on the assumption that the choice of immunization at a town hospital rather than at a village dispensary was based on town access.

### Ethics

Ethical clearance was granted by the Medical Research Co-ordinating Committee of the Tanzania National Institute for Medical Research, Tanzania. Shopkeepers and owners volunteered as informants after written informed consent, which guaranteed the confidentiality of information, anonymity and freedom to withdraw consent at any moment.

## Results

### Saleability

In-depth interviews with shopkeepers provided insight into their experience with selling drugs; which kinds of anti-malarials could be easily sold in Muheza and why?

#### Price

As expected, shopkeepers were aware that price was a determining factor for the kinds of drugs they could sell. The price of the cheapest ACT; a rare package of ALu (Coartem^®^) resold in a private shop, or more commonly a local brand of artesunate-sulfamethoxypyrazine-pyrimethamine, artesunate-SMP (Co-Arinate^®^), was four to seven times the cost of SP or SMP (Table 1). Generally the variety of drugs offered in a shop reflected its number of customers and likely added to its popularity. The most popular shops offered several kinds of ACT as alternatives to ALu, but with prices ranging up to 10,000 Tsh (8 US$), the saleability was low. The retail price of Coartem^® ^in the nearest licensed pharmacy (in Tanga) was 12,000 Tsh. Shop owners explained, that for as long as they were only allowed by the government to buy ALu at the retail price, it was impossible for them to sell it in Muheza. Only one shop offered ALu (Coartem^®^, two shops sold Co-Artesiane^®^, ALu suspension, but irregularly, and none had them at the time of the drug sale interviews. However, most shopkeepers said they had customers asking for ALu. If they came with a prescription for ALu, shopkeepers reported they could not change the prescription, but had to send them back. Only if they asked for ALu without a prescription, could customers be advised to buy e.g. *metakelfin *instead, as one shop-owner explained: "*they would agree then; people know the process (of waiting) at the hospital*". However, customers liked that ALu was free, or they only had to pay 0.4 US $ for it at the district mission hospital.

**Table 1:** Average anti-malarial drug prices in 16 private shops in Muheza

• Amodiaquine: 500 Tsh*
• SP/SMP: 700 Tsh
• AQ/SP syrups: 1,000-1,500 Tsh
• Quinine injectable: 3 ampuls 1,800 Tsh
• Quinine syrup for infant < 5 kg: 2,000 Tsh
• (Rarely available) ALu (Coartem^®^): 3,000 Tsh
• Artesunate-SMP (Co-Malafin^®^): 3,500- 5,000 Tsh
• Artesunate-SMP (Co-Arinate^®^) Junior: 7,500 Tsh
• ALu (Co-Artesiane^®^) syrup: 8,000 Tsh
• Artemisinin-naphtoquine (ARCO^®^): 8,000 Tsh
• Dihydroartemisinin-piperaquine (Duo-Cotexcin^®^): 10,000 Tsh

*1000 Tsh = 0.8 US $

#### Preferences

According to shopkeepers, their customers preferred the drugs they had become accustomed to after long time use: "*If they have used a drug since long time ago they do not want to change, they remember the name*". "*Each person has their own preference *(*pendekezo*)", was a common answer to why people bought the drugs they bought, or, "*these are his/her drugs*". Shopkeepers rarely felt the need to advice against the preferences of the customer. As one said, answering if he could sometimes advise people to use AQ he thought was stronger and better, instead of SP: "*It is their right to say "no thanks!" and say "then I prefer Fansidar^®^*".

Shopkeepers said that their customers appreciated single dose treatment for having side-effects for shorter time, and many preferred Metakelfin^® ^(SMP) to other SP, because it was only two pills instead of three, and hence perceived stronger and better. However, import of the brand Metakelfin^® ^had been banned recently following evidence that a fake product carrying its label was widespread at the market. Local or imported Kenyan SMP was however still widely available, all referred to as "*metakelfin*" regardless of actual brand name.

Answering if they had had customers coming to the shop after negative experiences with ALu, many shopkeepers said they had heard some people in the community saying they were getting fever again already the same month after taking the drug, and when they went to a clinic to be tested they were still malaria positive. Others stated they had received customers returning for *metakelfin *after ALu treatment to finish the cure, or who tried another drug than ALu for returning fever.

#### Side-effects

Evaluation of side-effects was said to be a major concern for consumers, when they selected between kinds or brands of anti-malarials. Shopkeepers said that their customers were concerned that SP could cause allergy (*mzio*), and dizziness (*kizunguzungu*) or headache (*kuumwa kichwa*) perceived to arise from the "sulpha" in SP. Such side-effects were said to vary with the quality of manufacturing, where Kenyan brands were often valued above Tanzanian ones, and imported Metakelfin^® ^had been very popular prior to the fake drugs scandal. Shopkeepers themselves were aware that SP and "*metakelfin" *were not alternative treatment options, but reported that many customers bought them interchangeable or later shifted to a Kenyan brand, to find out which they could tolerate best. Severe SP skin reactions were termed *babuka ngozi *(disfiguring the skin), explained as when a snake peels of its skin. One shopkeeper said he had seen a similar ALu skin reaction on TV. Customers, shopkeepers said, might also explain the seriousness of side effects or discomfort to rise if the diagnosis had been wrong (if they had taken anti-malarials but had no malaria). The term for medicine (*dawa*) is used also for poison, which might enhance popular expectations of discomfort during treatment. Some customers said that taking anti-malarials made them feel exhausted: "The drug weakens one (*zinachosha*), it is strong (*kali*)", and they feared to be unable to perform normal duties during the treatment. The shorter the duration, the better - hence the preference for SP. Some people were trying out merely different brands of the same drugs, to see which had least side-effects.

AQ was said often to cause nausea, dizziness and discomfort and was felt to lower blood pressure (*shusha presha *or *moyo kuenda chini*). Some customers nevertheless had AQ as "their drug"; shopkeepers explained that they were the ones who felt they had reactions from sulpha. Many shopkeepers explained the sensations of low blood pressure as lowered sugar in the blood, and said they advised their customers to take fruit or glucose to prevent this.

QN, mostly taken as syrup or injectables, was said to have worse side effects than AQ, including 'ringing in the ears' (*kelele masikioni*), but could easier be accepted as a strong yet necessary treatment, which people nevertheless were said to fear, because they knew it as the final treatment (formerly third-line, now second-line). Yet if they had got syrup for a child at the hospital an earlier time, they might continue to want that when their child got ill again, if they were satisfied with the drug. Shopkeepers said they sold QN on prescription, but differed in their opinion why health facilities were prescribing but not dispensing the drug. Some answered this was due to stock-outs at the hospital, others said people took a shortcut by not waiting at the hospital pharmacy before they could start treatment. When asked if they knew of people's experiences with ALu, although they were not themselves offering it for sale, shopkeepers told that ALu was a drug that did not have major side effects and people appreciated that.

### Sale

All 20 (100%) shops sold SP or sulphamethoxypyrazine-pyrimethamine (SMP), AQ and QN in oral children and adult formulations, QN also as injectable. CQ was sold in one shop, said to be for the malaria chemoprophylaxis for sickle cell disease patients (in accordance with national guidelines). Only half of the shops sold ACT, and none sold artemisinin mono-therapy. The calculated monthly sale of anti-malarials in the16 shops participating in the December drug sales survey was 4,041 doses, which if extrapolated to a full year, indicates that at any given month a quarter of the town population are self-medicating febrile illness with an anti-malarial bought from a private shop. The anti-malarial sale to the general population regardless of age was by far dominated by SP sale; brands of SP accounted for 74% of sales volume, equally divided between brands of SP and SMP (36% and 38% respectively). AQ accounted for 13%, and QN 11%, mainly as syrup or injectable. ACT accounted for only 2% of sale, mainly as Co-malafin^® ^(artesunate- SMP) or Duo-cotexcin^® ^(dihydroartemisinin -piperaquine). SP and AQ together (3,503 doses) would amount to the consumption of 2.8 doses of non-recommended anti-malarials per person per year.

**Figure 1:**
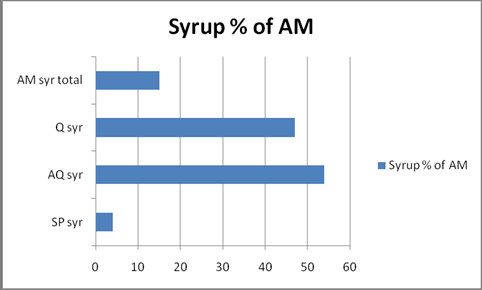
**Percentage of anti-malarial doses sold as syrup in 16 private drugshops in Muheza, Dec. 2009, Total 619 doses**.

At least 15% (619 doses) of drugs sale was to young children, based on the proportion of syrups of the total sale (Figure 1). Shops were providing 209 doses of quinine syrup monthly, sold in bottles containing only an adequate dose for an infant up to five kg, which is in accordance with guidelines when sold as first-line treatment. However, the insert drug information provides dosage regimes for children up to six years, without specifying that this is beyond the content, and infants are not weighed in drug shops. An estimated 1,412 infants in Muheza have access to shops in town; the number also brought to the town district hospital for immunization, rather than to village dispensaries (immunization coverage is high; 84% for diphtheria-tetanus-polio 3 (DTP) in Tanzania) [18]. The sale of quinine syrup from shops can be extrapolated to 2,508 infant doses annually; an estimated 1.8 dose pr. infant per year. This likely indicates, that at least one dose is taken when the infant is above five kilo, and the bottle will not last for a full one-week treatment.

**Figure 2:**
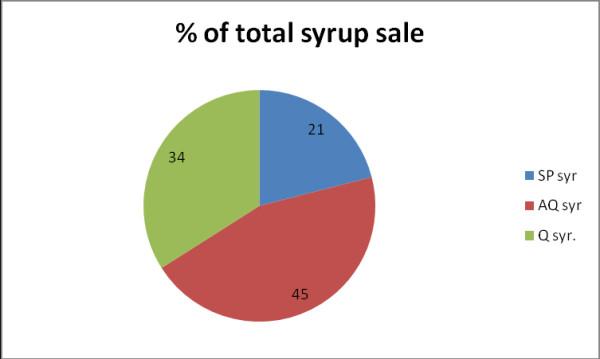
**Distribution of anti-malarial syrup sales volume in 16 private drugshops in Muheza, Dec. 2009, Total 619 doses**.

On top of the problem of a recommended drug (QN) sold at a non-recommended age, no less than 410 anti-malarial syrups were sold per month; 66% of syrup sale (Figure 2), was not quinine, but non-recommended anti-malarial, mainly AQ.

## Discussion

### High saleability of non-recommended anti-malarials

The continued private market for SP even after policy change is an interesting phenomenon, potentially with important lessons to be learned for the present and future anti-malarial drug policy changes. In the public sector, patients are not confronted with the choice of having to make evaluations of drug efficacy or "value for money". But the private sector offers that opportunity, and hence the demand for decision making, which is most often left to the customer, based on his/her knowledge and understanding of symptoms, ability and willingness to pay, and experiences and preferences of drugs. Evidently this leads to the purchase of cheap anti-malarials, even if no longer recommended in policy.

Locally, *malaria *is considered a form of *homa *(fever), and lay evaluations of drug efficacy is informed by cultural knowledge of illness [17]. If shopkeepers are right about their customers' views upon the drugs they sell - and the continued sale seems to support this - then people in Muheza still consider the cheapest drugs at the private market; AQ and SP (including SMP), to be efficacious drugs, that will keep them free from *homa *for long. According to shopkeepers, the vast majority of customers buying SP or *metakelfin *did so, because they found the drug was still working. The presence of this trust is supported by the magnitude of sale of these drugs - there is evidently still a market for anti-malarial mono-therapies in Muheza.

Although it can be argued that the alleged continued trust in the value of SP and AQ is informed by necessity - these are the sole drugs that are affordable to people - the alternative remains for them to obtain drugs from public facilities. As town dwellers with the option to chose between public and private sources of treatment, a substantial proportion of caretakers or patients in Muheza elected to seek care from drug shops, as shown below, in spite of the fact that here, ACT was 50% unavailable and 100% unsubsidized.

### The scale of private sale

Considering the size of the town; 15,000 residents, the study found a very high utilization of drug shops for the purchase of other anti-malarials, SP and SMP in particular, although these were officially replaced by ACT three years earlier. The large volume of anti-malarials sold in Muheza is surprising, considering that customers may often buy antipyretics without an anti-malarial as their first choice, and self-diagnose malaria only after antipyretic failure to lower fever [19]. However, observations of customers initially asking solely for antipyretics were rare - while these drugs were widely available in small general kiosks.

The private sector's share of the total dispensing of anti-malarials in the community is high, when compared with secondary data from the district hospital out-patient-department. The OPD at the District hospital Teule, the main source of primary health care for people in Muheza town, sees 31,075 new outpatients annually, among these 3,680 are children treated for malaria and/or anaemia [20]. Private drug shops provided an extrapolated 7,428 doses of anti-malarial syrup annually, i.e. more than double the OPD child malaria treatments at the hospital. Using the sale of anti-malarial syrups as a proxy for the proportion of anti-malarials sold to children is likely an underestimation; the price for syrup was double the price of tablets, and even if these could only be bought in pre-packaged adult doses they were a cheaper alternative. When at least two of every three child malaria treatments to non-hospitalised children are with drugs obtained from private shops, and two third of them are with non-recommended anti-malarials, it can be estimated that, unless admitted to hospital, almost half (44%) of Muhezan children treated for clinical malaria are not treated in accordance with national guidelines.

For the general population as for the children, care seeking outside the formal sector was frequent in private drug shops; the amount of anti-malarial drugs dispensed here accounted for an estimated three quarters of clinical malaria treatments in the community, as based on comparison with data [20] from the district hospital.

### Community SP drug pressure and resistance

Extrapolated to a year, private shops were providing each town inhabitant with almost three non-recommended anti-malarial doses per year; by far the majority of which would be with SP. This equals "*de facto *intermittent presumptive treatment", otherwise reserved for pregnant women. IPTp with SP is the only recommended treatment option to prevent maternal anaemia, placental malaria and adverse birth outcomes, as long as the safety of ACT for IPT during pregnancy remains unascertained.

High level of drug pressure in a community can rapidly lead to community-wide drug resistance, as has been shown for SP in Muheza already in 1994 [16]. A reduction in community drug resistance can be effected by known means, such as reducing the drug pressure by expanding the coverage of insecticide treated net ownership to the general population, not just to pregnant women and children [21,22], and, by ensuring the community's access and *use *of ALu, which is effective also in suppressing transmission [23].

SP drug resistance remains a reason for concern, for as long as SP is the only available recommended drug option for IPTp. The drug resistance data most recently published from Muheza come from clinical trials of IPTp, showing that IPTp use offered only partial protection and left those unprotected to suffer from a more intense infection, with increasing parasitaemia and a significantly higher fraction of highly resistant parasites as compared to infections in non-users of IPTp. The mean fraction of resistant parasites was 0.31 and increased by 0.15 each year from 2002 to 2005 [24]. A similar trend was found in nearby Korogwe, from ordinary treatment while SP was first-line drug. Here, a four-fold increase in the fraction of triple mutant resistance type parasites in infections was observed, from 8% in 2003 to 32% in 2007, along with a fivefold decline in parasite prevalence from 79% in 2003 to 15.5% in 2007 [25], indicating that the parasite pool for the transmission of drug resistant infection has remained relatively unaffected in size, in spite of the overall transmission decline.

Most recent data from the area (D. Ishengoma, personal communication) indicate that while presence of molecular markers reflecting CQ resistance may be declining, molecular markers for SP resistance remain high.

The present study adds to these findings, by demonstrating that the drug pressure in the community caused by the availability of SP in private drug shops even after a national drug policy change, equals *de facto *intermittent presumptive treatment not only by pregnant women, but by the community at large.

### Identified need for intervention

It is heartening that artemisinin mono-therapy, observed by the P.I. to be for sale in Muhezan drug shops in 2004 was no longer sold in any shops, indicating that a national ban since 2008 had been effective. However, prescription-only SP sales e.g. illicit sale remained high: 74% of total anti-malarial sale in private drug shops in Muheza. These data support the findings of a 2008-9 survey in other parts of Tanzania, showing that SP still accounted for three quarters of the sale of anti-malarials in private drug shops [26]. Even in Morogoro region, where the ADDO programme is already implemented, 65% of anti-malarial customers received SP, and only 13% received ALu [27]. The present study has shown that, although ACT is available in private shops, it is only accessed by a minority of the population. Increased access is blocked by lack of affordable recommended drugs at the private market, leaving the customers with older remedies, which they trust in and can afford and easily access from the private sector.

The persistence of a market for non-recommended drugs for self-medication risks undermining a public good; the efficacy of IPT for pregnant women. Policy and programme management of private distribution of anti-malarials are concerned with promoting shops' access to quality drugs to *dispense*. They must equally confront the problem how a parallel market for non-recommended drugs is effectively *dispensed with*. Training and accreditation of private drug dispensers is highly recommended, but should be followed up by studies on actual practice and performance.

### Study limitations

The observational study required the confidence of drug sellers working in an informal business sphere, where specialist drug stores unlicensed to sell prescription-only drugs nevertheless did so. This may have limited the sharing of confidential information. The question of the proportion of drug sale that was actually on prescription was left undiscovered for this reason. Sale without prescription could however be freely observed in all drug shops for the most commonly sold drugs.

With a singular focus on the private sector, the study only presents secondary data on the prescription practice in the public sector in Muheza, e.g. hospital statistics from the designated district hospital Teule, and only on diagnoses, not on treatments given [20]. As town inhabitants are free to seek care at the hospital OPD without prior referral, utilization of the public dispensary in Muheza town tends to be marginal, wherefore data missing from this facility would hardly have any impact.

The likely overlap in clients of the two kinds of facilities, hospital OPD patients and drug shop customers has not been established. Adjusting for any overlap would increase the relative proportion treated by private drug shops. Finally, it can only be assumed that purchased drugs are in fact consumed.

## Conclusions

An estimated three of every four cases of suspected malaria among the general population in Muheza were treated in the private sector, and almost nine of ten customers here obtained SP or AQ. Hence two of every three cases, considered as clinical malaria cases in the community, failed to get treated in accordance with national guidelines. The national malaria treatment policy reserves SP strictly for IPTp, and yet the drug is consumed almost thrice a year by the general population.

Considering the already very high level of drug resistance in the area, and the likelihood that a continuation of SP drug pressure will only aggravate the problem further, it is a major reason for concern that shops provide *de facto *intermittent presumptive treatment to the majority of the community. This is inappropriate treatment not only for the customers; it may compromise the strategy of IPT for pregnant women and render the safe SP use during pregnancy ineffective.

This might, in the end, reduce trust in and impact of the entire national malaria control. Training and accreditation - and supervision - of private drug dispensers is highly recommended.

## Competing interests

The authors declare that they have no competing interests.

## Authors' contributions

ICB and MML conceived of the study, FMR designed it and enrolled participants, FMR and ISM were each responsible for the implementation of the qualitative and quantitative study in the field, FMR analyzed data and drafted the manuscript. All authors reviewed the manuscript, and read and approved the final version.

## References

[B1] Alilio MS Kitua A Njunwa K Medina M Rønn AM Mhina J Msuya F Mahundi R Depinay JM Whyte S Krasnik A Bygbjerg IC Malaria control at the district level in Africa: the case of the Muheza district in northeastern Tanzania Am J Trop Med Hyg 2004 71 205 213 15331839

[B2] Goodman C Kachur SP Abdulla S Mwageni E Nyoni J Schellenberg JA Retail supply of malaria-related drugs in rural Tanzania: risks and opportunities Trop Med Int Health 2004 9 655 663 10.1111/j.1365-3156.2004.01245.x 15189455

[B3] Goodman C Kachur SP Abdulla S Bloland P Mills A Drug shop regulation and malaria treatment in Tanzania--why do shops break the rules, and does it matter? Health Policy Plan 2007 22 393 403 10.1093/heapol/czm033 17921151 PMC2657823

[B4] The Tanzania Food and Drugs Authority 2010 http://www.tfda.or.tz/addoprogramme.php

[B5] Ministry of Health and Social Welfare URoT National Guidelines for Diagnosis and Treatment of Malaria Malaria control series 11 2006 Dar es Salaam: National Malaria Control Program

[B6] World Health Organization WHO guidelines for the treatment of malaria 2006 Geneva: WHO/HTM/MAL/2006.1108

[B7] Alba S Hetzel MW Goodman C Dillip A Liana J Mshinda H Improvements in access to malaria treatment in Tanzania after switch to artemisinin combination therapy and the introduction of accredited drug dispensing outlets - a provider perspective Malar J 2010 9 164 10.1186/1475-2875-9-164 20550654 PMC2910018

[B8] Nsimba SE Rimoy GH Self-medication with chloroquine in a rural district of Tanzania: a therapeutic challenge for any future malaria treatment policy change in the country J Clin Pharm Ther 2005 30 515 519 10.1111/j.1365-2710.2005.00645.x 16336283

[B9] Nsimba SE How sulfadoxine-pyrimethamine (SP) was perceived in some rural communities after phasing out chloroquine (CQ) as a first-line drug for uncomplicated malaria in Tanzania: lessons to learn towards moving from monotherapy to fixed combination therapy J Ethnobiol Ethnomed 2006 2 5 10.1186/1746-4269-2-5 16403225 PMC1379633

[B10] Nsimba SE Assessing the performance, practices and roles of drug sellers/dispensers and mothers'/guardians' behaviour for common childhood conditions in Kibaha district, Tanzania Trop Doct 2007 37 197 201 10.1258/004947507782333099 17988473

[B11] Tarimo DS Minjas JN Bygbjerg IC Perception of chloroquine efficacy and alternative treatments for uncomplicated malaria in children in a holoendemic area of Tanzania: implications for the change of treatment policy Trop Med Int Health 2001 6 992 997 10.1046/j.1365-3156.2001.00818.x 11737836

[B12] Tarimo DS Malekela DA Health workers perceptions on chloroquine and sulfadoxine/sulfalene pyrimethamine monotherapies: implications for the change to combination therapy of artemether/lumefantrine in Tanzania East Afr J Public Health 2007 4 43 46 17907761

[B13] Eriksen J Nsimba SE Minzi OM Sanga AJ Petzold M Gustafsson LL Adoption of the new anti-malarial drug policy in Tanzania--a cross-sectional study in the community Trop Med Int Health 2005 10 1038 1046 10.1111/j.1365-3156.2005.01486.x 16185239

[B14] Wasunna B Zurovac D Goodman CA Snow RW Why don't health workers prescribe ACT? A qualitative study of factors affecting the prescription of artemether-lumefantrine Malar J 2008 7 29 10.1186/1475-2875-7-29 18252000 PMC2266770

[B15] United Republic of Tanzania The 2002 Population and Housing Census Results 2003 Dar es Salaam, Tanzania: Government Press

[B16] Rønn AM Msangeni HA Mhina J Wernsdorfer WH Bygbjerg IC High level of resistance of *Plasmodium falciparum *to sulfadoxine-pyrimethamine in children in Tanzania Trans R Soc Trop Med Hyg 1996 90 179 181 10.1016/S0035-9203(96)90129-7 8761583

[B17] Ringsted FM Bygbjerg IC Samuelsen H Early home-based recognition of anaemia via general danger signs, in young children, in a malaria endemic community in north-east Tan zania Malar J 2006 5 111 10.1186/1475-2875-5-111 17116250 PMC1676015

[B18] World Health Organization WHO/UNICEF Review of National Immunization Coverage - July 2009 http://apps.who.int/immunization_monitoring/en/globalsummary/countryprofileselect.cfm

[B19] Kamat VR Nyato DJ Community response to artemisinin-based combination therapy for childhood malaria: a case study from Dar es Salaam, Tanzania Malar J 2010 9 61 10.1186/1475-2875-9-61 20187949 PMC2842283

[B20] Teule Hospital DDH Muheza http://www.teule.or.tz/annual%20report%202007.pdf

[B21] Lemnge MM Alifrangis M Kafuye MY Segeja MD; Gesase S Minja D Massaga JJ Rønn AM Bygbjerg IC High reinfection rate and treatment failures in children treated with amodiaquine for falciparum malaria in Muheza villages, Northeastern Tanzania Am J Trop Med Hyg 2006 75 188 193 16896117

[B22] Alifrangis M Lemnge MM Rønn AM Segeja MD Magesa SM Khalil IF Bygbjerg IC Increasing prevalence of wildtypes in the dihydrofolate reductase gene of *Plasmodium falciparum *in an area with high levels of sulfadoxine/pyrimethamine resistance after introduction of treated bed nets Am J Trop Med Hyg 2003 69 238 243 14628937

[B23] Premji Z Gbadoe AD Marrast AC Gaye O Treatment of asymptomatic carriers with artemether-lumefantrine: an opportunity to reduce the burden of malaria? Malar J 2011 10 64 10.1186/1475-2875-10-64 20096111 PMC2824802

[B24] Harrington WE Mutabingwa TK Muehlenbachs A Sorensen B Bolla MC Fried M Competitive facilitation of drug-resistant *Plasmodium falciparum *malaria parasites in pregnant women who receive preventive treatment Proc Natl Acad Sci USA 2009 106 9027 9032 10.1073/pnas.0901415106 19451638 PMC2690058

[B25] Alifrangis M Lusingu JP Mmbando B Dalgaard MB Vestergaard LS Ishengoma D Five-year surveillance of molecular markers of *Plasmodium falciparum *antimalarial drug resistance in Korogwe District, Tanzania: accumulation of the 581G mutation in the *P. falciparum *dihydropteroate synthase gene Am J Trop Med Hyg 2009 80 523 527 19346369

[B26] President' Malaria Initiative (PMI) FY Malaria Operational Plan (MOP) Tanzania 2010 http://www.fightingmalaria.gov/countries/mops/fy10/tanzania_mop-fy10.pdf

[B27] Alba S Dillip A Hetzel MW Mayumana I Mshana C Makemba A Improvements in access to malaria treatment in Tanzania following community, retail sector and health facility interventions - a user perspective Malar J 2010 9 163 10.1186/1475-2875-9-163 20550653 PMC2910017

